# Prevalence and Risk Factors of Insomnia and Sleep-aid Use in Emergency Physicians in Japan: Secondary Analysis of a Nationwide Survey

**DOI:** 10.5811/westjem.2022.12.57910

**Published:** 2023-02-20

**Authors:** Takuyo Chiba, Yusuke Hagiwara, Toru Hifumi, Yasuhiro Kuroda, Shunya Ikeda, Danya Khoujah, Takahiro Imaizumi, Takashi Shiga

**Affiliations:** * International University of Health and Welfare, Department of Emergency Medicine, Narita, Chiba, Japan; † International University of Health and Welfare, Graduate School of Medicine, Minatoku, Tokyo, Japan; ‡ Tokyo Metropolitan Children’s Medical Center, Department of Pediatric Emergency Medicine and Critical Care Medicine, Tokyo, Japan; § St. Luke’s International Hospital, Department of Emergency and Critical Care Medicine, Tokyo, Japan; ¶ University of Maryland School of Medicine, Department of Emergency Medicine, Baltimore, Maryland; || Kagawa University, Faculty of Medicine, Kita, Kagawa, Japan; # University of Maryland Upper Chesapeake Medical Center, Department of Emergency Medicine, Bel Air, Maryland; ** Nagoya University Hospital, Department of Advanced Medicine, Nagoya, Aichi, Japan

## Abstract

**Introduction:**

Emergency physicians (EP) are suspected to have a high prevalence of insomnia and sleep-aid use. Most prior studies about sleep-aid use in EPs have been limited by low response rates. In this study our aim was to investigate the prevalence of insomnia and sleep-aid use among early-career Japanese EPs and assess the factors associated with insomnia and sleep-aid use.

**Methods:**

We collected anonymous, voluntary, survey-based data regarding chronic insomnia and sleep-aid use from board-eligible EPs taking the initial Japanese Association of Acute Medicine board certification exam in 2019 and 2020. We describe the prevalence of insomnia and sleep-aid use and analyzed demographic and job-related factors using multivariable logistic regression analysis.

**Results:**

The response rate was 89.71% (732 of 816). The prevalence of chronic insomnia and sleep-aid use was 24.89% (95% CI 21.78–28.29%) and 23.77% (95% CI 20.69–27.15%), respectively. Factors associated with chronic insomnia were long working hours (odds ratio [OR] 1.02, 1.01–1.03, per one-hour/week), and “stress factor” (OR 1.46, 1.13–1.90). Factors associated with sleep-aid use were male gender (OR 1.71, 1.03–2.86), unmarried status (OR 2.38, 1.39–4.10), and “stress factor” (OR 1.48, 1.13–1.94). The “stress factor” was mostly influenced by stressors in dealing with patients/families and co-workers, concern about medical malpractice, and fatigue.

**Conclusions:**

Early-career EPs in Japan have a high prevalence of chronic insomnia and sleep-aid use. Long working hours and stress were associated with chronic insomnia, while male gender, unmarried status, and stress were associated with the use of sleep aids.

## INTRODUCTION

Emergency physicians’ (EP) sleep cycles and wellness are challenged by their irregular shift work.^1^ Night shifts are associated with sleep difficulty, shorter sleep time, low levels of alertness, and less optimal performance by emergency medicine (EM) residents and attendings.^2^ Driving after night shifts is related to a high risk of motor vehicle collisions and near-crashes for EM residents.^3^ Shift work in general is also associated with increased risk of stroke and coronary heart disease.^4^,^5^ Sleep disturbance is linked to burnout,^6^ and an American College of Emergency Physicians Policy Resource and Education Paper reported that the adverse effect of rotating shifts is the most important reason for early attrition from EM.^7^

Despite these detrimental effects, night shifts will always be a part of the EP’s work routine in order to provide the necessary care for patients and maintain the essential functions of hospitals. Due to shift work and the resulting sleep disturbance. insomnia and sleep-aid use appear to be prevalent among EPs.^8^–^12^ In fact, some North American studies report that as many as 56% of EPs use a sleep aid.^13^ However, most of the data about insomnia and sleep-aid use in EPs is based on surveys limited by low response rates.^8^–^10^ The prevalence of insomnia and sleep-aid use is not known among Japanese EPs but is presumed to be higher than the reported national Japanese average of 12.2–21.5%.^14^,^15^ Nevertheless, these reported numbers are alarmingly high, especially when considered in relation to the possible harmful effects of sleep-aid use, such as impaired sleep quality, daytime somnolence, and psychomotor impairment.^16^ In addition, factors associated with insomnia and sleep-aid use have not been well studied.

In this study our goal was to describe the prevalence of insomnia and sleep-aid use among early-career EPs in Japan, as well as identify the factors associated with chronic insomnia and sleep-aid use. Understanding the factors associated with insomnia and sleep-aid use may contribute to changes in the way EPs work to reduce the need for the use of sleep aids for insomnia, as well as a heightened awareness of this problem in those who are innately high risk.

## METHODS

### Study Design and Population

This study is a secondary analysis of the survey-based career satisfaction data collected in 2019 and 2020 from board-eligible EPs taking the Japanese Association for Acute Medicine (JAAM) initial board certification exam. To become board-eligible in EM in Japan, physicians must complete a two-year general, combined medical-surgical transitional training program, in addition to at least three years of EM training at an accredited residency program. Emergency medicine residents in Japan spend most of their time in the emergency department (ED) to experience a predetermined number of procedures and cases, but there are no specific rules regarding how many months they need to spend in the ED or the sequence of these rotations. They also spend a fair number of rotations in the intensive care unit (ICU). Upon graduating residency, EPs in Japan often work in both the ED and ICU. The majority of physicians practicing in the ICU are also board-certified in EM,^17^ and the majority of those who are not board-certified in critical care are EPs.^18^ The details of creating the career-satisfaction survey and survey results were published in 2021.^19^ Printed questionnaires were distributed to the exam participants and were completed at the site anonymously and voluntarily. The questionnaire included questions about the following domains: demographics (age, gender, postgraduate year, marital status, presence of children in their life); work environment; insomnia, burnout, and use of sleep aids; professional satisfaction; and concerns and stressors. All questions were multiple choice (Table 1).

**Population Health Research Capsule**

What do we already know about this issue?

*Shift work is an unavoidable element of emergency medicine, Insomnia and sleep-aid use are reported to be common among emergency physicians (EP)*.

What was the research question?

*We aimed to describe the prevalence and cause of their insomnia and sleep-aid use based on a survey of Japanese EPs who took the board certification exam in 2019 and 2020*.

What was the major finding of the study?

*The prevalence of chronic insomnia and sleep-aid use were 24.89% and 23.77%, respectively. Stress and long working hours were associated factors*.

How does this improve population health?

*Improvements in work style and alleviation of stress may positively affect the well-being of EPs*.

### Data Analysis

The current study focuses on insomnia and sleep-aid use. With regard to insomnia, the participants selected an answer from four choices: had never experienced insomnia; had a brief episode of insomnia; had an experience of chronic insomnia; or currently suffered from chronic insomnia. For statistical analysis, we considered the prevalence of insomnia in our population to include those respondents whose answer was either “had an experience of chronic insomnia” or “currently suffered from chronic insomnia.” No specific definition was included to delineate chronic from brief insomnia. As for sleep-aid use, we asked the respondents to select all the sleep aids they had used (if any) from six different categories: alcohol; antihistamine; benzodiazepine; herbal supplement; analgesic; and other. Given that melatonin is not available as an over-the-counter medication in Japan, it was not included as a separate choice. Questions about the time and frequency of sleep-aid use were not asked.

Regarding Likert-type response items, we first conducted factor analysis. Factor analysis was performed to identify a small number of more meaningful latent factors behind many variables in an attempt to explain the data using this small number of factors. In factor analysis, we included 16 questions that asked about professional satisfaction as well as four questions about concerns and stressors—all graded on a five-point Likert scale. We reviewed floor effects and ceiling effects for each question before conducting factor analysis. The number of factors extracted was decided based on the scree plot of eigenvalues and the previous study.^19^

In the factor analysis, we used maximum likelihood with the expectation-maximization algorithm to estimate the covariance matrix for missing data.^20^,^21^ We then performed multivariable logistic regression analysis with factor scores obtained through the factor analysis and other demographic and work environment data including gender, age, marital status, postgraduate year (PGY), working hours per week, presence of children in the home, long shifts (longer than 24-hour shift to cover nights), number of night shifts per month, annual number of ambulances arriving at the facility, and number of attending EPs at the facility. Postgraduate year was viewed as a dichotomous value: 5 to 7, or ≥8. Before we performed the multivariable logistic regression analysis, missing data was imputed 20 times with multiple imputation by chained equation, and the results were combined applying Rubin’s rule. We performed statistical analysis with complete dataset for sensitivity analysis. We used Stata16 (StataCorp LLC, College Station, TX) for statistical analysis. A *P*-value less than 0.05 was considered statistically significant.

### Ethical Consideration

All data was collected anonymously, and this survey was approved by the JAAM Ethics Committee. (JAAM20180808)

## RESULTS

**Figure 1:**
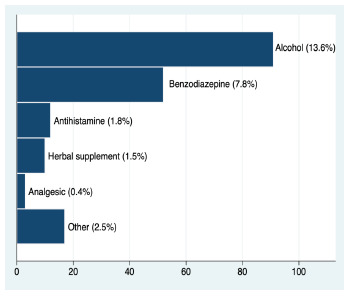
Sleep-aid use pattern.

The questionnaire was handed out to 433 and 383 board-eligible EPs at the board exam in 2019 and 2020, respectively. Response rates were 93.07% in 2019 (403 of 433) and 85.90% in 2020 (329 of 383), and the overall response rate was 89.71% (732 of 816). The majority of respondents were in their thirties (77.15%), with a median of PGY-7 (6.0–10.0). One-third of the participants were PGY-10 or greater (269 of 732). Of 679 respondents, 169 (24.89%, 95% confidence interval [CI] 21.78%–28.29%) reported that they experienced chronic insomnia, while 159 of 669 respondents reported a history of sleep-aid use (23.77%, 95% CI 20.69%–27.15 %) (Table 2). Sixty-one participants reported both chronic insomnia and a history of sleep-aid use. Alcohol was the most common sleep-aid; 91 reported the use of alcohol (13.62%, 95% CI 11.22%–16.44%). Benzodiazepine was the second most reported sleep-aid (Figure 1).

We performed factor analysis after confirming there were no floor effects or ceiling effects. We extracted four factors with maximum likelihood method and subsequently performed Promax rotation. A professional satisfaction question regarding patient volume at current facility did not show sufficient factor loading; therefore, it was removed from further analysis. We performed factor analysis again with maximum likelihood method and Promax rotation with the expectation-maximization algorithm for missing data. These extracted factors were inductively categorized as the following: “educational/clinical system factor”; “work condition factor”; “skill/knowledge development factor”; and “stress factor,” all of which were based on items that had high factor loading for each factor (Table 3).

Stress in dealing with patients/families and co-workers, concern about medical malpractice, and fatigue had high factor loading for the “stress factor” category. With regard to multiple imputation, the number of incomplete data that required imputation were 37–134 of 732 total participants (5.32%–22.41%) (Data Supplement 1). The proportion of missing data was the highest in the factors extracted in the factor analysis, and the lowest in gender. Multivariable logistic regression analysis revealed that chronic insomnia was associated with long working hours, and the “stress factor”; the ORs were 1.02 (95% CI 1.01–1.03, per one-hour/week), and 1.46 (95% CI 1.13–1.90), respectively (Table 4). Sleep-aid use was associated with male gender, unmarried status, long shift (longer than 24 hours shift to cover nights), and the stress factor; the ORs were 1.71 (95% CI 1.03–2.86), 2.38 (95% CI 1.39–4.10), 1.86 (95% CI 1.22–2.85), and 1.48 (95% CI 1.13–1.94), respectively (Table 5).

Complete case analysis without imputation for missing data showed that in addition to long working hours and stress, male gender was inversely associated with chronic insomnia (Data Supplement 2). Regarding sleep-aid use, male gender, unmarried status, and stress were associated with sleep-aid use in the complete case analysis. In the complete case analysis, long shifts showed a trend toward increased sleep-aid use but did not reach statistical significance (OR 1.64, 95% CI 0.99–2.66) (Data Supplement 3). Variance inflation factors for items included in the multivariable logistic regression analyses were less than 2.2 and did not suggest the existence of multicollinearity.

## DISCUSSION

This nationwide survey of early-career EPs in Japan during their initial emergency medicine (EM) board certification exam showed that approximately one-fourth of EPs had experienced chronic insomnia and one-fourth had tried sleep aids for their insomnia. Factors associated with chronic insomnia were long working hours and stress. Factors associated with sleep-aid use were male gender, unmarried status, and stress.

Previous studies have shown that insomnia and sleep-aid use among EPs worldwide is common. Mail-based and web-based studies on United States (US) EM residents and Canadian EPs reported the use of sleep-aid was 34.2–46.2%.^8^–^10^ Unfortunately, these surveys had low response rates (16–49.6%), thereby limiting the ability to generalize this data and estimate the true rate of sleep-aid use in EPs. In 2014, a similar survey of US allopathic EM residents with a high response rate (72 %) found that 71% used chemical aids to stay awake or go to sleep.^11^ This higher rate of chemical aids was most likely due to inclusion of stimulants such as coffee or energy drinks. Furthermore, a 2006 nationwide study in the US focusing on EM residents of various levels of training revealed a 21.8% prevalence of past zolpidem use, with 9.3% reporting recent use.^22^

A 2019 web-based survey of EPs working in five EDs in Calgary, Canada, with a high response rate (73%), reported a rate of 56% of current pharmacologic sleep-aid use (95% CI 48–64%), which was significantly higher than previous studies. This may be due in part to a higher average age of respondents than prior studies, as more than half the participants were above the age of 39.^13^ Most of these studies focused on EM residents; studies on practicing EPs post-training are limited. The issue of sleep-aid use is not limited to North American EPs. A web-based survey from Saudi Arabia reported that 36.6% of EPs, paramedics, and EM technicians use sleep-aids, and that an increase in use was associated with a higher average number of monthly night shifts.^12^

Another voluntary, anonymous, online cross-sectional study in Australia reported that 46.5% of EPs used medications such as melatonin, benzodiazepine, and pseudoephedrine to manage their sleep and performance.^23^ The response rate of these web-based studies is unclear due to unknown denominators. A study based on the Taiwanese National Health Insurance Research Database reported that the prevalence of insomnia was 5.56% and the percentage of hypnotic use was 19.96%.^24^ This result was based on *International Classification of Diseases* codes and the prescription data recorded in the national database; it did not include self-treated insomnia or sleep-aid use without prescription. Therefore, it most likely underestimated the rate of insomnia and sleep-aid use.

Our study reveals a comparable prevalence of insomnia and sleep-aid use to the aforementioned international studies. This reported prevalence of insomnia is higher than the age-matched general Japanese population. A study conducted in 1997 on the general Japanese population showed the prevalence of insomnia in those aged 30–39 was 15.95%, while a 2008 study reported the prevalence of insomnia in the same age group was 11.7% in males and 10.3% in females.^14^,^15^ The sleep-aid use in this age group appears to be 2.1–2.6%.^14^ Moreover, it is possible that EPs in Japan may be more reluctant to use a sleep aid or report the use of sleep aids due to cultural views of sleep disturbance and substance use. Albeit a possible underestimation, these reported numbers are alarmingly high, especially considering that the participants are still early in their careers, with a presumed long career and subsequent stressors ahead of them. It is highly unlikely that our reported results were high only due to the participants’ recent residency training, as one-third of them were PGY-10 or higher.

Several studies reported the prevalence of sleep-aid use in EPs, but there is little data about risk factors associated with insomnia and sleep-aid use. Understanding the factors associated with insomnia and sleep-aid use would lead to further studies regarding interventions to improve EPs’ sleep. Although it was a subjective report, participants in a 2004 study suggested that work hours, demands of work, emotional stress from work-related activities, family commitments, and changing circadian rhythms were causing fatigue and difficulty in initiating sleep.^9^ Another study found that the average monthly number of night shifts was associated with sleep-aid use.^12^ Our study suggests that chronic insomnia is associated with long working hours and stress, and sleep-aid use is associated with stress, after excluding some non-modifiable factors.

We often discuss the need to limit the working hours and number of night shifts, and the importance of modifying the EP’s work style (fewer working hours per week and shorter shifts) is further strengthened by the results of this study. It is worth noting that more than 90% of study participants reported working more than 40 hours a week, and more than 30% of them reported working longer than 24 hours to cover nights. There is no official recommendation regarding shift length for EPs in Japan; the length of shifts reported in this study seems much longer than recommended in France and the US.^25^,^26^ In addition to work style, we also need to focus on physicians’ stressors at the workplace to improve EPs’ sleep cycles, as this study showed that the stress factor is associated with both chronic insomnia and sleep-aid use in Japanese EPs. Reducing the stress of EPs may improve the prevalence of insomnia or sleep-aid use.

## LIMITATIONS

This study has some limitations. First, we collected this data from relatively young Japanese EPs. Those who have been practicing in EM longer may show a different pattern from the results we obtained in this study. The participants’ recent intense EM residency training might have affected the results. The fact that all of them had just taken their board exam might have created additional stress for the participants, thus affecting the results. In addition, the results may not be applicable to EPs outside Japan whose cultural background and work styles are different from those within Japan. However, the previous studies showed similar patterns in other countries, and we think it is reasonable to estimate that EPs have a high prevalence of insomnia and sleep-aid use.

Secondly, survey-based data has some inherent biases, such as selection bias, measurement bias, and subject bias. The EPs who responded to the survey may have different sleep patterns and sleep-aid use habits from those who did not respond. However, the high response rate nearing 90% minimizes the possibility that this bias affected our study results. The subjective definition of insomnia by the participants may have affected the results. It is also not clear how many of the respondents who answered the questionnaire reported the truth due to social-desirability bias. Some people may feel that reporting experiencing insomnia could be perceived as a sign of weakness, and others may have felt a reluctance to report their use of sleep aids, thereby lowering the reported prevalence of insomnia and sleep-aid use. This bias was addressed by keeping responses anonymous and participation voluntary. Finally, due to the observational nature of the study, we could not determine any causal relationship between the factors listed above and insomnia or sleep-aid use, but rather an association.

## CONCLUSION

This secondary analysis of a nationwide survey showed that Japanese emergency physicians have a high prevalence of insomnia and sleep-aid use early in their careers. Alcohol and benzodiazepine were the two most commonly used sleep aids reported. In addition to demographic background and work style such as longer work hours per week, stress was associated with chronic insomnia and sleep-aid use. Further studies are needed to investigate what intervention can improve EPs’ sleep hygiene.

## Supplementary Information



## Figures and Tables

**Table 1:** The survey items.

Basic information							
(1) What is your gender?	○ Male		○ Female				
(2) What is your age?							
(3) Postgraduate year	○ PGY6		○ PGY7	○ PGY8	○ PGY9		○ PGY>10
(4) Are you married?	○ Married		○ Unmarried				
(5) Do you have a child (children)?	○ Yes		○ No				
(6) Choose your type of practice	○ ED only		○ ED combined with inpatient ward, ICU, and/or OR				
(7) Choose your type of night shift.							
A: Night shifts are always connected with day shifts. (Works longer than 24 hours to cover nights.)							
B: Night shifts are separated from day shifts.							
C: Mixture of A and B							
(8) Number of night shifts per month	○ 1–3		○ 4–5	○ 6–7	○ 8–9		○ ≥10
(9) Working hours per week	○ ≤40		○ 40–59	○ 60–79	○ 80–100		○ ≥101
(10) Monthly salary (unit: 10000 yen)	○ ≤30		○ 31–50	○ 51–70	○ 71–100		○ ≥101
(11) Number of ambulance transfers per year							
○ ≤2000	○ 2001–4000		○ 4001–6000	○ 6001–8000	○ 8001–10000		○ ≥10001
(12) Number of attending emergency physicians (board-certified) at your facility							
○≤2	○3–6		○≥7				
Insomnia and Burnout							
(1) What is your experience of insomnia?							
○ Never experienced insomnia	○ Had a brief episode of insomnia						
○ Had an experience of chronic insomnia	○ Currently suffer from chronic insomnia						
(2) Which substance have you used as sleep-aid?							
○ Never used sleep-aid		○Alcohol		○ Antihistamine		○ Benzodiazepine	
○ Herbal supplement		○ Analgesic		○ Others			
(3) What is your experience of burnout							
○ No symptoms of burnout	○ Occasionally I am under stress, but I don’t feel burned out.						
○ Definitely burning out and have symptoms of burnout, such as physical and emotional exhaustion							
○ The symptoms of burnout that I’m experiencing won’t go away.							
○ Completely burned out and often wonder if I can go on.							
Professional Satisfaction (5-point Likert scale: 1, very dissatisfied; 5, very satisfied)							
Personal							
(1) How satisfied are you with your income?							
(2) How satisfied are you with your private time?							
(3) How satisfied are you with your knowledge in emergency medicine?							
(4) How satisfied are you with your development of skills and knowledge through practice?							
(5) How satisfied are you with the opportunity to participate in conference?							
(6) How satisfied are you with your time reading medical literature to learn new knowledge?							
Residency Program							
(7) How satisfied are you with how organized your residency training was?							
(8) How satisfied are you with the number of supervising attending physicians during training?							
(9) How satisfied are you with the bedside education you received from attending physicians?							
(10)Overall, how satisfied are you with your emergency medicine residency training?							
Current Facility							
(11) How satisfied are you with the access to clinical resources for problem solving?							

Professional Satisfaction (5-point Likert scale: 1, very dissatisfied; 5, very satisfied)							

(12) How satisfied are you with the availability of teaching opportunities in your current position?							
(13) How satisfied are you with the availability of research opportunities in your current position?							
(14) How satisfied are you with the emergency department management by administrators?							
(15) How satisfied are you with the patient volume?							
(16) How satisfied are you with working hours?							
Overall satisfaction							
(17) How satisfied are you with your emergency medicine career?							
Concerns and Stressors (5-point Likert scale: 1, low; 5, high)							
(1) How concerned are you about medical malpractice?							
(2) How stressed are you from issues associated with patients and their families?							
(3) How stressed are you from issues associated with your colleague, including nurses, pharmacists, radiology technicians, and administrators?							
(4) How fatigued are you?							
Career Satisfaction (Yes/No)							
(1) Do you have a mentor(s)?							
(2) If you were to go back to a time before emergency medicine residency, would you still choose emergency medicine again as your specialty?							
(3) Would you switch your specialty?							

*ED*, emergency department; *ICU*, intensive care unit; *OR*, operating room; *PGY*, postgraduate year.

**Table 2:** Participants’ characteristics.

		Total	Chronic insomnia (−)	Chronic insomnia (+)	P-value	Total	Sleep-aid (−)	Sleep-aid (+)	P-value
		N=679	N=510	N=169		N=669	N=510	N=159	
Male gender		540 (79.5%)	410 (80.4%)	130 (76.9%)	0.32	531 (79.6%)	400 (78.7%)	131 (82.4%)	0.37
Age	20–29	30 (4.5%)	25 (5.0%)	5 (3.0%)	0.08	30 (4.5%)	26 (5.2%)	4 (2.5%)	0.58
	30–39	520 (77.5%)	399 (79.0%)	121 (72.9%)		509 (76.9%)	388 (77.0%)	121 (76.6%)	
	40–49	103 (15.4%)	68 (13.5%)	35 (21.1%)		104 (15.7%)	75 (14.9%)	29 (18.4%)	
	50–59	10 (1.5%)	6 (1.2%)	4 (2.4%)		11 (1.7%)	9 (1.8%)	2 (1.3%)	
	≥60	8 (1.2%)	7 (1.4%)	1 (0.6%)		8 (1.2%)	6 (1.2%)	2 (1.3%)	
Post-graduate year	6–7	351 (52.2%)	277 (54.9%)	74 (44.0%)	0.02	346 (52.2%)	270 (53.4%)	76 (48.4%)	0.31
	≥8	322 (47.8%)	228 (45.1%)	94 (56.0%)		317 (47.8%)	236 (46.6%)	81 (51.6%)	
Unmarried		209 (30.8%)	159 (31.2%)	50 (29.6%)	0.70	211 (31.6%)	141 (27.7%)	70 (44.0%)	<0.01
With child		332 (50.1%)	249 (50.0%)	83 (50.3%)	1.00	325 (49.7%)	261 (51.8%)	64 (42.7%)	0.05
Shift length ≥ 24 hours		208 (31.0%)	148 (29.3%)	60 (36.4%)	0.10	203 (30.7%)	144 (28.5%)	59 (37.8%)	0.03
Working hours per week	40	28 (4.2%)	23 (4.6%)	5 (3.0%)	<0.01	26 (4.0%)	18 (3.6%)	8 (5.1%)	0.64
	41–60	261 (39.2%)	212 (42.2%)	49 (29.9%)		254 (38.7%)	196 (39.4%)	58 (36.7%)	
	61–80	240 (36.0%)	180 (35.9%)	60 (36.6%)		239 (36.4%)	179 (35.9%)	60 (38.0%)	
	81–100	94 (14.1%)	66 (13.1%)	28 (17.1%)		93 (14.2%)	74 (14.9%)	19 (12.0%)	
	≥101	43 (6.5%)	21 (4.2%)	22 (13.4%)		44 (6.7%)	31 (6.2%)	13 (8.2%)	
Night shifts per month	1–3	104 (15.7%)	74 (14.9%)	30 (18.1%)	0.03	100 (15.2%)	79 (15.8%)	21 (13.5%)	0.37
	4–5	234 (35.2%)	177 (35.5%)	57 (34.3%)		231 (35.2%)	172 (34.4%)	59 (37.8%)	
	6–7	204 (30.7%)	166 (33.3%)	38 (22.9%)		202 (30.8%)	148 (29.6%)	54 (34.6%)	
	8–9	83 (12.5%)	56 (11.2%)	27 (16.3%)		84 (12.8%)	70 (14.0%)	14 (9.0%)	
	≥10	39 (5.9%)	25 (5.0%)	14 (8.4%)		39 (5.9%)	31 (6.2%)	8 (5.1%)	

**Table 3:**
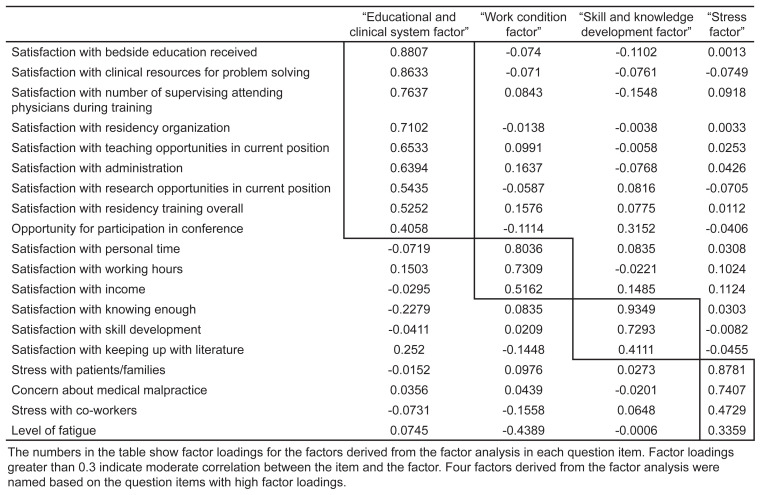
Factor loading of questionnaire items for inductively categorized factors (pattern matrix after Promax rotation).

**Table 4:** Multivariable logistic regression analysis for chronic insomnia.

Risk factor for chronic insomnia	Odds ratio (95% CI)	P-value
Male gender	0.64 (0.40 – 1.03)	0.06
Age	1.02 (0.98 – 1.06)	0.26
Unmarried	0.78 (0.46 – 1.32)	0.35
Child	0.89 (0.54 – 1.46)	0.64
Long shift	1.34 (0.88 – 2.04)	0.18
Working hours per week	1.02 (1.01 – 1.03)	<0.01*
Night shifts per month	1.00 (0.92 – 1.01)	0.92
“Educational and clinical system factor”	1.07 (0.80 – 1.43)	0.67
“Work condition factor”	0.76 (0.55–1.04)	0.08
“Skill and knowledge development factor”	0.92 (0.70–1.21)	0.56
“Stress factor”	1.46 (1.13 – 1.90)	<0.01*
Postgraduate year	1.27 (0.83 – 1.95)	0.27
Annual ambulance number	1.00 (1.00 – 1.00)	0.77
Attending number	1.00 (0.88 – 1.12)	0.96

* *P* < 0.05.

*CI*, confidence interval.

**Table 5:** Multivariable logistic regression analysis for sleep-aid use.

Risk factor for sleep-aid use	Odds ratio (95% CI)	P-value
Male gender	1.71 (1.03 – 2.86)	0.04*
Age	1.02 (0.98 – 1.06)	0.39
Unmarried	2.38 (1.39 – 4.10)	<0.01*
Presence of children	0.89 (0.50 – 1.56)	0.68
Long shift	1.86 (1.22 – 2.85)	<0.01*
Working hours per week	0.99 (0.98 – 1.01)	0.22
Night shifts per month	0.96 (0.87 1.06)	0.44
“Educational and clinical system factor”	0.84 (0.62 – 1.12)	0.23
“Work condition factor”	0.98 (0.72 – 1.34)	0.92
“Skill and knowledge development factor”	1.15 (0.88 – 1.51)	0.31
“Stress factor”	1.48 (1.13 – 1.94)	<0.01*
Postgraduate year	1.35 (0.86 – 2.12)	0.19
Annual ambulance number	1.00 (1.00 – 1.00)	0.44
Attending number	1.13 (1.00 – 1.28)	0.06

* *P* < 0.05.

*CI*, confidence interval.
