# Supplementary material for: Prevalence and Risk Factors of Insomnia and Sleep-aid Use in Emergency Physicians in Japan: Secondary Analysis of a Nationwide Survey
Source: West J Emerg Med. 2023 Feb 20;24(2):331–9. doi: 10.5811/westjem.2022.12.57910 (PMC10047722; doi:10.5811/westjem.2022.12.57910)
Supplement: Supplementary file 1 [file wjem-24-331-s001.docx]

| Data Supplement 1. Data requiring imputation | | |
| --- | --- | --- |
| Covariates (N = 732) | Complete | Incomplete |
| Male sex | 695 | 37 (5.32%) |
| Age | 687 | 45 (6.55%) |
| Unmarried | 694 | 38 (5.48%) |
| Child | 677 | 55 (8.12%) |
| Long shift | 684 | 48 (7.02%) |
| Working hours per week | 678 | 54 (7.96%) |
| Night shifts per month | 677 | 55 (8.12%) |
| Educational/Clinical system factor | 598 | 134 (22.41%) |
| Work condition factor | 598 | 134 (22.41%) |
| Skill/Knowledge development factor | 598 | 134 (22.41%) |
| Stress factor | 598 | 134 (22.41%) |
| Post-graduate year | 690 | 42 (6.09%) |
| Annual ambulance number | 650 | 82 (12.62%) |
| Attending number | 632 | 100 (15.82%) |
| Chronic insomnia | 679 | 53 (7.81%) |
| Sleep-aids use | 669 | 63 (9.42%) |

| Data Supplement 2. Multivariable logistic regression analysis for chronic insomnia (complete case analysis) | | |
| --- | --- | --- |
| Covariates | Odds Ratio (95%CI) | p-value |
| Male sex | 0.42 (0.24- 0.74) | <0.01* |
| Age | 1.04 (0.99 - 1.08) | 0.12 |
| Unmarried | 0.57 (0.31 - 1.05) | 0.07 |
| Presence of children | 0.75 (0.42 - 1.34) | 0.34 |
| Long shifts | 1.00 (0.61 - 1.63) | 1.00 |
| Working hours per week | 1.02 (1.01 - 1.04) | <0.01* |
| Night shifts per month | 1.00 (0.90 - 1.12) | 0.94 |
| Educational and clinical system factor | 1.00 (0.72 - 1.39) | 1.00 |
| Work condition factor | 0.91 (0.65 - 1.29) | 0.60 |
| Skill and knowledge development factor | 0.78 (0.54 - 1.12) | 0.18 |
| Stress factor | 1.41 (1.04 - 1.91) | 0.03* |
| Post-graduate year | 1.14 (0.68 - 1.89) | 0.63 |
| Annual ambulance number | 1.00 (1.00 - 1.00) | 0.85 |
| Attending number | 0.94 (0.82 - 1.08) | 0.38 |

| Data Supplement 3. Multivariable logistic regression analysis for sleep-aid use (complete case analysis) | | |
| --- | --- | --- |
| Covariates | Odds Ratio (95%CI) | p-value |
| Male sex | 2.04 (1.08 – 3.88) | 0.03* |
| Age | 0.99 (0.94 - 1.03) | 0.61 |
| Unmarried | 2.65 (1.39 - 5.08) | <0.01* |
| Presence of children | 1.06 (0.56 - 2.01) | 0.86 |
| Long shifts | 1.62 (0.99 -2.66) | 0.05 |
| Working hours per week | 0.99 (0.98 - 1.01) | 0.32 |
| Night shifts per month | 0.91 (0.81- 1.03) | 0.12 |
| Educational and clinical system factor | 0.81 (0.58 -1.13) | 0.21 |
| Work condition factor | 1.14 (0.81 - 1.60) | 0.45 |
| Skill and knowledge development factor | 0.94 (0.65 - 1.35) | 0.73 |
| Stress factor | 1.38 (1.02 - 1.87) | 0.04* |
| Post-graduate year | 1.34 (0.80- 2.24) | 0.26 |
| Annual ambulance number | 1.00 (1.00 - 1.00) | 0.17 |
| Attending number | 1.11 (0.96 - 1.27) | 0.16 |
